# The Effects of Exercise Order on the Psychophysiological Responses, Physical and Technical Performances of Young Soccer Players: Combined Small-Sided Games and High-Intensity Interval Training

**DOI:** 10.3390/biology10111180

**Published:** 2021-11-15

**Authors:** Ersan Arslan, Bulent Kilit, Filipe Manuel Clemente, Yusuf Soylu, Mustafa Sögüt, Georgian Badicu, Firat Akca, Mine Gokkaya, Eugenia Murawska-Ciałowicz

**Affiliations:** 1 Faculty of Sport Sciences, Tokat Gaziosmanpasa University, Tokat 60250, Turkey; ersanarslan1980@hotmail.com (E.A.); yusuf.soylu@gop.edu.tr (Y.S.); 2 School of Physical Education and Sports, Tekirdag Namik Kemal University, Tekirdag 59030, Turkey; bkilit@nku.edu.tr; 3 Escola Superior Desporto e Lazer, Instituto Politécnico de Viana do Castelo, Rua Escola Industrial e Comercial de Nun’Álvares, 4900-347 Viana do Castelo, Portugal; filipeclemente@esdl.ipvc.pt; 4 Department of Physical Education and Sports, Middle East Technical University, Ankara 06800, Turkey; msogut@metu.edu.tr; 5 Department of Physical Education and Special Motricity, University Transilvania of Brasov, 500068 Brasov, Romania; georgian.badicu@unitbv.ro; 6 Faculty of Sport Sciences, Ankara University, Ankara 06560, Turkey; fakca@ankara.edu.tr (F.A.); minegokkaya@ankara.edu.tr (M.G.); 7 Physiology and Biochemistry Department, University School of Physical Education, 51-612 Wroclaw, Poland

**Keywords:** soccer, high-intensity, small-sided games, psychophysiological responses, combined training

## Abstract

**Simple Summary:**

Small-sided games are very popular training methods, among other commonly used strategies, for enhancing the functional and sport-specific skills of young soccer players. In addition, high-intensity interval training has the potential to increase the aerobic capacity of youths. No study has compared the order effects of combined small-sided games and high-intensity interval training on the physical performances, psychophysiological responses, and technical skills of young soccer players. The results of this research show practical information that can help to design training programmes for youth soccer players.

**Abstract:**

This study aimed to compare the order effects of combined small-sided games (SSGs) and high-intensity interval training (HIIT) on the psychophysiological responses and physical and technical performances of young soccer players. Twenty-four soccer players (aged 14.63 ± 0.71 years) were randomly divided into SSGs + HIIT (*n* = 12) and HIIT + SSGs (*n* = 12) for 6 weeks. The SSGs consisted of two 4–16 min rounds of 2, 3, and four-a-side games with 2 min of passive resting, whereas the HIIT consisted of 6–10 min of high-intensity runs at varying intensities (from 90 to 100%). Pre-test and post-test elements included a 5–30 m sprint test, countermovement jump test, zigzag agility test with the ball and without the ball, repeated sprint ability test, speed dribbling ability test, three-corner run test, and Yo-Yo Intermittent Recovery Test level 1. Both combined training interventions produced similar improvements in physical performance and technical responses (*p* ≥ 0.05, *d* values ranging from 0.40 to 1.10). However, the combined HIIT + SSGs training produced meaningfully lower perceived exertion (*p* = 0.00, *d* = 2.98) and greater physical enjoyment (*p* = 0.00, *d* = 4.28) compared with the SSGs + HIIT intervention. Furthermore, the SSGs + HIIT group showed a higher training load than those from the HIIT + SSGs group for all weeks (*p* ≤ 0.05, *d* values ranging from 1.36 to 2.05). The present study’s results might be used by coaches and practitioners to design training programmes for youth soccer players.

## 1. Introduction

High-level performances in soccer are combined with physical performance, psychophysiological responses, and technical abilities during small-sided games [1,2,3]. Therefore, several alternative training methods to traditional ones have been proposed to enhance the physical and technical capabilities of young soccer players. High-intensity interval training (HIIT), one of the increasingly popular training modalities, is defined as intense and intermittent exercises interspersed with recovery periods [4]. It requires a reduced amount of time and thus allows young athletes greater time to train their sport skills [5]. Earlier studies have documented the positive influences of HIIT on various physical fitness parameters [6] and soccer-specific performance characteristics in young soccer players [7].

Small-sided games (SSGs)—training strategies that are more enjoyable, effective, and time-efficient—are another commonly used method for training the functional and sport-specific skills of young soccer players [1,8]. SSGs, which are derived from street soccer and are played with fewer players, smaller pitch areas, and modified rules [9,10], simultaneously involve actual game dynamics, technical and tactical skills, and physical demands under changeable game conditions [8,11]. Consequently, some studies have shown the contribution of SSGs to aerobic fitness, repeated sprint ability, linear sprinting, agility, change of direction, and jumping performance in young players [1,12].

A recent systematic review demonstrated the effectiveness of combined HIIT and SSGs for soccer players [13]. As a result of this study, it was discovered that combining SSGs and running-based training methods induced higher external and internal load values and greater improvements in overall fitness capacity compared to the intervention using only SSGs. On the other hand, the researchers found a larger improvement in aerobic fitness for professional players who only participated in SSGs when compared to players who participated in combined training [14]. The inconsistency among these aforementioned studies shows that more research is needed to understand the efficiency of combined training.

Several studies recently compared the effects of combined game-based and HIIT programmes in team sports [13,15]. While some coaches routinely use the combined SSGs and HIIT approach (starting with SSGs and then performing HIIT or the opposite) to optimise sport-specific technical and tactical learning without any physiological or psychological fatigue effect on performance, others may prefer the combined HIIT and SSGs approach to have players undertake game performance, including technical and tactical tasks, under fatigue conditions [16,17]. The mechanisms related to running-based and game-based training are naturally different, although both tax aerobic and anaerobic metabolisms. Running-based HIIT seems to elicit a greater proportion of anaerobic metabolism. Blood lactate concentrations vary between 4 and 9 mmol/L in short/long HIIT [6], while SSGs vary between 0.5 and 4 mmol/L [18]. Moreover, neuromuscular effects are also different. Short HIIT or nonmaximal efforts produce peripheral fatigue (e.g., alterations to muscle excitability and excitation–contraction coupling), while SSGs can produce more variability based on the type of stimulus occurring in a match [19]. Therefore, it can be expected that starting with one type of HIIT over another might constrain the physiological responses, which would interfere with the next method. In a pioneering study, researchers examined the influences of combined training with different exercise orders on semi-professional soccer players [20]. Their results indicated that changing exercise orders yielded a similar enhancement in intermittent fitness performance. However, to the best of the authors’ knowledge, there are no additional data on the impacts of combination order on multiple performance parameters in soccer players. Therefore, the aim of the present study was to compare the order effects of combined SSGs and HIIT on the psychophysiological responses and physical and technical performances of young soccer players.

## 2. Materials and Methods

### 2.1. Study Design

A two-group, matched, experimental design was used in the present study. The study was completed over a total of 9 weeks, consisting of 1.5 weeks of pre-testing, 6 weeks of combined training interventions (SSGs + HIIT or HIIT + SSGs), and 1.5 weeks of post-testing. The players completed a 30–15 intermittent fitness test (30–15 IFT), speed dribbling ability (SDA) test, 5–30 m sprint test, countermovement jump (CMJ) test, repeated sprint ability (RSA) test, zigzag agility test with the ball (ZAWB) and without the ball (ZAWOB), three-corner run test (TCRT), and Yo-Yo Intermittent Recovery Test level 1 (YYIRT-1) before and after the 6-week combined intervention period. Both training interventions were performed twice a week and each daily training session was separated by a minimum of 2 days to avoid fatigue-induced adverse effects. During the present study, the players performed the same type of daily training, and combined training interventions were added to their training sessions. After 15 min of standardised warmup, which consisted of jogging and dynamic stretching at each training session, players performed combined training, including SSGs + HIIT or HIIT + SSGs. All tests and training sessions with the same order were performed on a natural grass soccer pitch.

### 2.2. Subjects

Twenty-four young male soccer players participated in the present study. The players were separated into two combined groups: the SSGs + HIIT group (*n* = 12, age: 14.67 ± 0.65 years) and the HIIT + SSG group (*n* = 12, age: 14.58 ± 0.79 years). All players were also members of the U-16 regional amateur league teams. They were accustomed to a training workload of ≥3 training units per week, consisting of core strength, plyometric and technical drills, and had been involved in soccer training and competitive soccer matches for at least 2 years. Before the study, all players and their parents were fully informed about the procedures to be used and completed voluntary written consent forms. The study was performed in accordance with the Declaration of Helsinki and the Research Ethics Committee of the local university.

### 2.3. Procedures

Testing Procedure. On the first day, to calculate body fat percentage, the skinfold thickness technique was used with a Holtain Tanner–Whitehouse skinfold calliper (Holtain, UK) before breakfast. Skinfold thickness was measured twice at each site and the mean of two measurements was used to calculate body fat percentage. Body fat percentage was calculated using the equation that has been validated for males aged 15 to 24 years in young Turkish athletes [21]. After anthropometric measurements, determination of individual players’ high-intensity intermittent running performance with changes in direction was assessed using the 30–15 IFT. The test, which consists of 30 s of running and 15 s of passive recovery, is a reliable progressive field test according to the procedures performed by Buchheit [22]. On the third day, the SDA test was used for the evaluation of soccer-specific technical skills and according to procedures described by Rosch et al. [23]. Briefly, the test, which is available in the F-MARC test battery designed by FIFA, allows for the assessment of coordinated speed dribbling under time pressure. After the technical test, each player performed three straight 30 m sprint test (5 m, 10 m, and 20 m splits) performances with 2 min of passive resting.

On the fifth day, each player was tested on their vertical jump height using the CMJ test according to the procedures performed by Arslan et al. [1]. A portable force plate (Newtest, Finland) was used to assess the CMJ test performances. Following the CMJ test, each player performed 6 repetitions of a 30 m maximal sprint with a 180° change of direction (15 m + 15 m). Twenty seconds of recovery were allowed between shuttle sprints [24]. The ZAWB and ZAWOB tests were performed to evaluate the agility performances of the players on the seventh day. The test, which included soccer-specific movement patterns [25], consisted of four 5 m sections with each change of trajectory angled at 100° as reported by Mirkov et al. [26]. The TCRT was performed to assess the speed endurance and anaerobic endurance of the players on a natural grass pitch [23]. The running times in these tests were measured using a timing gate photocell system. We found high test–retest reliability (ICC = >0.86) for tests such as sprinting, jumping, agility, and technical skill. On the ninth day, to evaluate maximum oxygen consumption (VO_2max_), the YYIRT-1, which is an acoustically progressive field test [27], was performed according to procedures explained by Bangsbo et al. [28]. After the test, the estimated VO_2max_ was calculated using the following formula:

VO_2max_ = 36.4 + (0.0084 × covered distance in YYIRT-level 1)

Training Interventions. The training procedure is summarised in Table 1. During the 6-week training period, young players performed 2 combined training sessions (SSGs + HIIT or HIIT + SSGs) a week in addition to their 3 days of soccer-specific training. Their weekly training routine consisted of 5 60–75 min practice sessions and 1 soccer match. During the study, their coach generally focused on developing core strength and technical and tactical skills, except for the 2 combined training sessions. After 15 min of standardised warmup, which consisted of jogging and dynamic stretching at each training session, players performed combined training, including SSGs + HIIT or HIIT + SSGs. A gradual progress plan was designed to reach maximal final performance in combined training programmes. Players performed 2, 3, and 4-a-side formats of SSGs, including free game, possession, and small goal for two 4–16 min games per training session according to the procedures detailed by Sanchez-Sanchez et al. [29]. Verbal encouragements were given by coaches throughout the SSGs. Players performed HIIT sessions, which consisted of 15 s of intermittent running at 90–100% of players’ velocity at IFT (VIFT), followed by 15 s of resting (Table 1).

The rating of perceived exertion (RPE) was obtained using the category ratio scale (6–20) to calculate the internal training load (ITL) immediately after the completion of each session [30]. The scale was introduced at the beginning in order to familiarise the players. All players also completed a short form of the physical activity enjoyment scale (PACES). This scale includes 5 items scored on a 1–7 Likert scale and has been validated [31] as a marker of enjoyment level for physical activity by Turkish youth [1].

### 2.4. Statistical Analyses

Data were expressed as mean ± standard deviation (SD). Group differences in psychophysiological responses, in terms of RPE, PACES, and ITL (overall) results between SSGs + HIIT and HIIT + SSGs, were assessed using the independent sample *t*-test. A mixed ANOVA was used to test for interactions and main effects for time (pre- vs. post-test) and group (SSGs + HIIT vs. HIIT + SSGs) on the physical and technical performances. Effect sizes (Cohen’s *d*) were also calculated for each dependent variable. Cohen’s *d* values were considered trivial (<0.20), small (0.20–0.59), moderate (0.6–1.19), large (1.2–1.99), and very large (≥2.0) [32]. All statistical analyses were computed using SPSS version 24.0 (SPSS, Version 24.0 for Windows; SPSS Inc., Chicago, IL, USA). Statistical significance was set at the level of *p* ≤ 0.05.

## 3. Results

Pre-test values and the effect of combined training on the body composition, physical performance responses, and technical skills of the players are summarised in Table 2.

**Figure 1:**
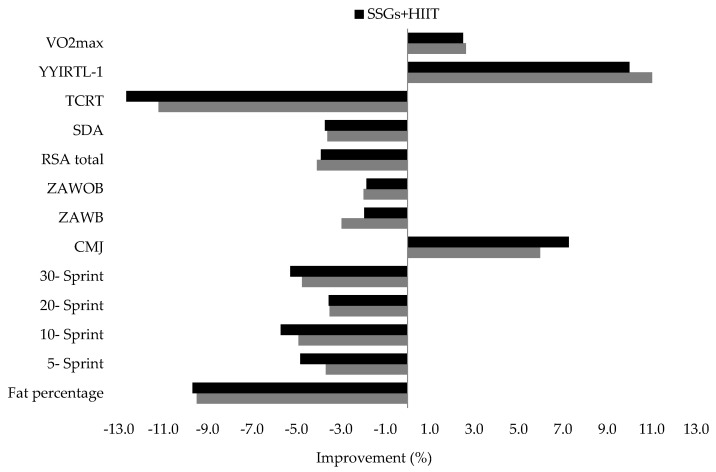
Improvement in body composition, physical and technical performance responses following the combined training interventions.

Both combined training interventions (SSGs + HIIT and HIIT + SSGs) showed similar improvements in body composition, physical performance responses, and technical skills (*p* ≥ 0.05, *d* values ranging from 0.40 to 1.10) (Table 2) (Figure 1).

**Figure 2:**
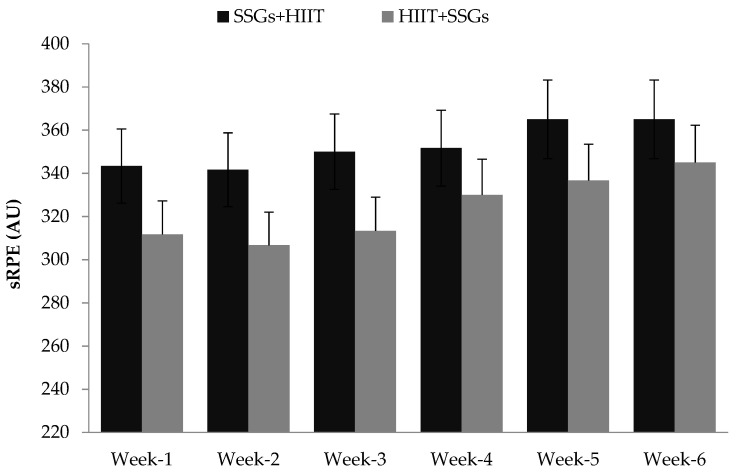
Weekly internal training loads during the 6 weeks combined training interventions.

Overall RPE responses to HIIT + SSGs training were meaningfully lower than those from the SSGs + HIIT group (16.2 ± 0.5 vs. 17.6 ± 0.5; *p* = 0.00, *d* = 2.98). Moreover, overall PACES scores from the HIIT + SSGs training were meaningfully greater than those from the SSGs + HIIT group (30.7 ± 1.1 vs. 26.3 ± 0.9; *p* = 0.00, *d* = 4.28). Conversely, the SSGs + HIIT group demonstrated a higher training load than those from the HIIT + SSGs group for all weeks (*p* ≤ 0.05, *d* = ranging from 1.36 to 2.05) (Figure 2).

## 4. Discussion

The aim of this study was to analyse the effects of exercise order in a combined training programme including SSGs and HIIT. The results of this parallel study revealed no significant differences between groups (SSGs + HIIT vs. HIIT + SSGs) in the fitness measures collected after the 6-week intervention. However, both combined programmes revealed significant pre–post improvements in linear sprinting, agility, vertical jump, aerobic capacity, and repeated-sprint ability.

A combination of SSGs and running-based HIIT was recently tested, aiming to provide the advantageous effect of running-based HIIT to the training programmes based on SSGs [12]. The first reported combination of SSGs and HIIT revealed the beneficial effect of the combination compared to a group using just SSGs for the improvement of VO_2max_ and 30–15 VIFT [15]. However, they did not consider how to implement the combination.

Exercise order is of paramount importance. In the first study, testing the effects of exercise order within a training session [20], it was revealed that no significant differences were found between those who completed SSGs + HIIT and those who completed HIIT + SSGs in the 30–15 VIFT [20], which revealed that the internal load imposed was similar between groups. In the present study, the measures of aerobic capacity (i.e., YYIRTL-1 and VO_2max_) were both meaningfully improved by 6-week training interventions, with no significant difference considering the exercise order. Those findings are not surprising, since both SSGs and running-based HIIT have been repeatedly confirmed as effective in improving aerobic capacity [33,34]. The capacity to sustain high efforts while using both SSGs and running-based HIIT ensures that cardiorespiratory and aerobic systems are taxed by the training stimulus, thus promoting beneficial adaptations [35]. The nonexistence of differences between exercise order is in line with the previous work [20] and suggests that exercise intensity can be independent of the order of implementation.

Considering the effects of combined training intervention on linear sprinting, it was surprising to observe meaningful improvements independent of the exercise order, considering previous reports of combined SSGs + HIIT on such physical quality [14,15]. In fact, the results of the present study showed significant improvements of both groups in the 5, 10, 20, and 30 m sprint, thus suggesting the effectiveness of implementing SSGs and HIIT to improve linear sprinting. This fact was not observed in a previous study that combined SSGs and endurance and speed training or in the study that combined SSGs and short-interval (15′–15′) HIIT [15]. In fact, recent systematic reviews with meta-analysis revealed inconsistences and the ineffectiveness of SSGs [12] and HIIT [34] in improving linear sprinting in soccer players. One possible reason for observing improvements in the current research is due to the age effect and the capacity for improvement in this sensitive period [36].

Change of direction (COD) and agility with the ball were both capacities elicited equally by the combination of SSGs + HIIT with no difference considering the exercise order. The use of SSGs and HIIT independently has been suggested as a good way to improve COD [37], while SSGs seem to be better at improving agility with the ball compared to HIIT [38,39]. The combination of both in the present study contributed to meaningfully improving COD and agility with the ball, independent of the exercise order. Again, it seems that exercise order does not affect the capacity of both programmes and training methods to promote beneficial effects in these skills. However, it seems important to highlight the beneficial effect of combining HIIT with regular SSGs, considering a recent meta-analysis that suggested a significant favourable effect of HIIT in comparison to SSGs in improving linear sprint and COD in a within-group analysis [12]. This may be caused by the limited capacity of performing high-intensity linear or curvilinear running activities in small spaces as in the case of SSGs. On the other hand, those small spaces in SSGs can be helpful for promoting greater stimulus in COD and agility with the ball [40].

RSA was also improved after both combined interventions in which exercise order had no significant effect. Recent meta-analysis on the effects of HIIT in soccer revealed a significant favourable effect on RSA [34] in which no significant differences occurred between using HIIT or SSGs [41]. Therefore, due to the specific intermittence and energetic systems associated with both SSGs and HIIT, meaningful improvements in this capacity would be expected [42,43]. Improvements in lower-limb power and sprinting may also be factors benefiting the improvements in RSA [44]. In fact, in the current study, CMJ was also significantly improved in both combined interventions, thus suggesting possible positive effects, despite not being in line with recent meta-analysis about the use of HIIT and SSGs in soccer [12,34] and also compared with a study that combined SSGs + HIIT [45]. It is possible that the age effect and window of improvement may have caused the improvements observed in the current study.

Regarding the consequences of different exercise orders on psychophysiological responses and training load, it was found that HIIT + SSGs had meaningfully lower values of RPE, training load, and enjoyment than the group of SSGs + HIIT. The results regarding the training load are not in line with a previous study that tested the same issue (SSGs vs. HIIT and vice versa), in which no meaningful differences were found [20]. In this case, the psychological effect of more enjoyment while playing SSGs may have affected the perception of load. In fact, consistent results revealed that SSGs induce greater enjoyment than running-based HIIT [1,8,46]. Thus, for the groups ending the session with HIIT, the combination of fatigue and the least enjoyable activity may play an important role in the perceived effort and the enjoyment reported.

This study had some limitations. No control group was implemented; thus, it is not possible to compare the evolution of players without a training intervention or with different training interventions. Additionally, age may be a constraint for the possible generalisation of the findings since the study was conducted in a critical period of evolution. Future studies should compare combined interventions with single training or alternative training methods. Additionally, extending the research to more age groups and normalising the maturation status would be interesting.

## 5. Conclusions

The present study showed the order effects of combined SSGs and HIIT on the psychophysiological responses and physical and technical performances of young soccer players. After 6 weeks of combined training interventions, both combined training groups demonstrated similar improvements in physical performance and technical responses. However, the effects of exercise order demonstrated meaningful differences in psychophysiological responses and training load. In terms of practical implications, this study suggests that the combination of SSGs + HIIT is effective in improving the fitness status of adolescent soccer players. However, exercise order does not seem to have a determinant effect on the consequences of the changes in fitness. Therefore, coaches may organise the order based on the most appropriate plans. Future applications should consider implementing strength training in addition to the combination of SSGs + HIIT.

## Figures and Tables

**Table 1:** Description of the 6 weeks of combined training programs.

Week	Sessions	Game Formats	Pitch Dimension	SSGs+HIIT	HIIT+SSGs
				**Pre-Intervention Testing**	
1	1	2 v 2	15 × 27	2 × (2 × 2 min FG), 2 min rest 2 × (5 min of 15″-15″ at 90% of V_IFT_)	2 × (5 min of 15″-15″ at 90% of V_IFT_) 2 × (2 × 2 min FG), 2 min rest
	2	3 v 3	20 × 30	2 × (3 × 3 min FG), 2 min rest 2 × (4 min of 15″-15″ at 90% of V_IFT_)	2 × (4 min of 15″-15″ at 90% of V_IFT_) 2 × (3 × 3 min FG), 2 min rest
2	3	4 v 4	25 × 32	2 × (4 × 4 min FG), 2 min rest 2 × (3 min of 15″-15″ at 90% of V_IFT_)	2 × (3 min of 15″-15″ at 90% of V_IFT_) 2 × (4 × 4 min FG), 2 min rest
	4	2 v 2	15 × 27	2 × (2 × 2 min POS), 2 min rest 2 × (5 min of 15″-15″ at 90% of V_IFT_)	2 × (5 min of 15″-15″ at 90% of V_IFT_) 2 × (2 × 2 min POS), 2 min rest
3	5	3 v 3	20 × 30	2 × (3 × 3 min POS), 2 min rest 2 × (4 min of 15″-15″ at 90% of V_IFT_)	2 × (4 min of 15″-15″ at 90% of V_IFT_) 2 × (3 × 3 min POS), 2 min rest
	6	4 v 4	25 × 32	2 × (4 × 4 min POS), 2 min rest 2 × (3 min of 15″-15″ at 90% of V_IFT_)	2 × (3 min of 15″-15″ at 90% of V_IFT_2 (4 × 4 min POS), 2 min rest
4	7	2 v 2	15 × 27	2 × (2 × 2 min SG), 2 min rest 2 × (5 min of 15″-15″ at 95% of V_IFT_)	2 × (5 min of 15″-15″ at 95% of V_IFT_) 2 × (2 × 2 min SG), 2 min rest
	8	3 v 3	20 × 30	2 × (3 × 3 min SG), 2 min rest 2 × (4 min of 15″-15″ at 95% of V_IFT_)	2 × (4 min of 15″-15″ at 95% of V_IFT_) 2 × (3 × 3 min SG), 2 min rest
5	9	4 v 4	25 × 32	2 × (4 × 4 min SG), 2 min rest 2 × (3 min of 15″-15″ at 95% of V_IFT_)	2 × (3 min of 15″-15″ at 95% of V_IFT_) 2 × (4 × 4 min SG), 2 min rest
	10	2 v 2	15 × 27	2 × (2 × 2 min FG), 2 min rest 2 × (5 min of 15″-15″ at 100% of V_IFT_)	2 × (5 min of 15″-15″ at 100% of V_IFT_) 2 × (2 × 2 min FG), 2 min rest
6	11	3 v 3	20 × 30	2 × (3 × 3 min POS), 2 min rest 2 × (4 min of 15″-15″ at 100% of V_IFT_)	2 × (4 min of 15″-15″ at 100% of V_IFT_) 2 × (3 × 3 min POS), 2 min rest
	12	4 v 4	25 × 32	2 × (4 × 4 min SG), 2 min rest 2 × (3 min of 15″-15″ at 100% of V_IFT_)	2 × (3 min of 15″-15″ at 100% of V_IFT_) 2 × (4 × 4 min SG), 2 min rest
				Post-intervention testing	

FG: free game; POS: possession; SG: small goal; V_IFT_: Maximum speed reached in the last stage of the 30-15 Intermittent Fitness Test.

**Table 2:** Effect of both training methods on physical and technical performances of the participants.

	SSGs + HIIT (*n* = 12)			HIIT + SSGs (*n* = 12)			Training Comparison		
	Pre-Test	Post-Test	Change	Pre-Test	Post-Test	Change	F_(1, 22)_	p	η^2^
Body fat (%)	9.78 ± 2.53	8.83 ± 2.32 *	−0.95	9.48 ± 1.57	8.58 ± 1.49 *	−0.90	0.112	0.741	0.005
5-m (s)	0.95 ± 0.05	0.90 ± 0.06 *	−0.05	0.93 ± 0.06	0.90 ± 0.06 *	−0.03	0.157	0.696	0.007
10-m (s)	1.68 ± 0.05	1.58 ± 0.06 *	−0.10	1.62 ± 0.05	1.54 ± 0.08 *	−0.08	3.737	0.066	0.145
20-m (s)	3.06 ± 0.11	2.95 ± 0.10 *	−0.11	3.05 ± 0.20	2.94 ± 0.21 *	−0.11	0.048	0.829	0.002
30-m (s)	4.42 ± 0.09	4.19 ± 0.08 *	−0.23	4.34 ± 0.26	4.14 ± 0.23 *	−0.20	0.861	0.364	0.038
CMJ (cm)	31.72 ± 2.70	33.98 ± 2.44 *	2.26	32.01 ± 2.10	33.90 ± 1.89 *	1.89	0.013	0.911	0.001
ZAWB (s)	8.44 ± 0.32	8.28 ± 0.31 *	−0.16	8.60 ± 0.23	8.40 ± 0.31 *	−0.20	1.479	0.237	0.063
ZAWOB (s)	6.92 ± 0.23	6.79 ± 0.23 *	−0.13	6.79 ± 0.36	6.66 ± 0.37 *	−0.13	1.083	0.309	0.047
RSA_total_ (s)	39.08 ± 1.01	37.55 ± 0.93 *	−1.53	38.68 ± 1.08	37.09 ± 0.73 *	−1.59	1.297	0.267	0.056
SDA (s)	25.90 ± 1.47	24.94 ± 1.49 *	−0.96	25.00 ± 1.53	24.09 ± 1.39 *	−0.91	2.113	0.160	0.088
TCRT (s)	28.63 ± 0.47	25.01 ± 0.95 *	−3.62	28.32 ± 1.10	25.14 ± 0.98 *	−3.16	0.066	0.800	0.003
YYIRTL-1 (m)	1248.3 ± 107.7	1393.0 ± 107.1 *	144. 7	1213.3 ± 95.5	1363.3 ± 87.7 *	150.0	0.816	0.376	0.036
VO_2max_ (mL.min^−1^.kg^−1^)	46.89 ± 0.90	48.10 ± 0.90 *	1.21	46.59 ± 0.80	47.85 ± 0.74 *	1.26	0.816	0.376	0.036

* *p* ≤ 0.05 for within-group changes.

## Data Availability

Not applicable.
